# Nonequal-length image encryption based on bitplane chaotic mapping

**DOI:** 10.1038/s41598-024-58612-8

**Published:** 2024-04-20

**Authors:** Ruqing Zhang, Rigui Zhou, Jia Luo

**Affiliations:** School of Information Engineering, Shanghai Maritime University, Shanghai, 201306 China

**Keywords:** Computer science, Information technology

## Abstract

In recent years, extensive research has focused on encryption algorithms for square images, with relatively little attention given to nonsquare images. This paper introduces a novel encryption algorithm tailored for nonequal length images, integrating bit-plane chaotic mapping and Arnold transformation. To effectively implement the algorithm, the plain image is initially transformed into two equal-sized binary sequences. A new diffusion strategy is then introduced to mutually diffuse these sequences, followed by the use of a chaotic map to control the swapping of binary elements between them, enabling permutation of bits across different bitplanes. Finally, the positional information of the image is scrambled using the Arnold transform, resulting in the generation of the encrypted image. By utilizing nonequal Arnold transformation parameters and the initial value of the Lorenz chaotic map as keys, the transmission of keys is simplified, and the cryptosystem gains infinite key space to resist brute force attacks. Experimental results and security analysis confirm the effectiveness of the proposed quantum image encryption algorithm in encrypting nonsquare images, demonstrating good performance in terms of nonstatistical properties, key sensitivity, and robustness. Furthermore, simulation experiments based on Qiskit successfully validate the correctness and feasibility of the quantum image encryption algorithm.

## Introduction

With the development of the Internet, communication methods are constantly evolving and becoming more closely intertwined with people's lives. The Internet has generated a vast amount of information data, with images as the primary carrier, accounting for over 75% of the data. Within this vast image data, there is a wide range of information, including public data generated through normal browsing and publishing on the internet, as well as private information that involves personal privacy. With the increasing demand for image protection, the field of image encryption has emerged, attracting the attention and research of numerous scholars^1^.

Quantum image processing technology plays a significant role in ensuring image security, and it can be realized through the utilization of quantum computers^2^. To store and capture images for subsequent quantum image encryption operations, a series of quantum image representation models have been proposed^3–5^. In 2005, Latorre et al.^6^ put forward the realistic Ket model which utilizes the properties of quantum superposition but is not conducive to quantum image processing. In 2010, Le et al.^7^ introduced the Flexible Representation of Quantum Images (FRQI) model, which is suitable for global image transformations and offers high processing speed. However, it is not suitable for local image transformations and does not allow for precise image measurements. The following year, Sun et al.^8^ extended FRQI to the RGB color space and proposed the MCRQI model, which utilizes three quantum bits to store color information and opacity information for RGB. In 2013, Zhang et al.^9^ introduced the NEQR model, which addresses the issue of accurately measuring grayscale information in FRQI within a limited number of operations. In 2014, Li et al.^10^ proposed the Normal Arbitrary Superposition State (NASS) model, which can represent multi-dimensional images without requiring additional qubits to store color information. Building on NEQR, Jiang et al.^11^ introduced the Generalized Quantum Image Representation (GQIR) model in which image sizes can be arbitrary integers, and the number of qubits needed to store the image is reduced. In 2017, Sang et al.^12^ presented a novel representation method called New Color Quantum Image (NCQI), which utilizes three quantum registers to represent the three color channels of a color digital image. Additionally, it reduces the time complexity in the image preparation process. Also in the same year, Yao et al.^13^ proposed the Quantum Probability Image Encoding (QPIE) model, which allows encoding of rectangular images. However, this model faces challenges in accurately extracting the original image from its encoded quantum circuit.

Several image encryption algorithms have been proposed based on these representations models of quantum images. In the field of encryption technology based on chaos theory, chaotic systems are considered to be the most suitable for image encryption due to their characteristics of unpredictability, ergodicity, and sensitivity to initial values. Since the publication of the first work on chaotic encryption by Matthew^14^, researchers have proposed numerous encryption schemes based on chaos theory. In 1998, Fridrich introduced parameters into two-dimensional chaotic maps, generalized and discretized them into finite square grids, and then extended them to three-dimensional space, creating a symmetric block encryption scheme^15^. In 2004, in order to improve the algorithm's resistance against statistical analysis and differential attack analysis, Chen et al.^16^ first extended the two-dimensional chaotic cat map to three dimensions, utilizing the positional information of the three-dimensional map to achieve good interference effects. In order to achieve fast image encryption, Kwok et al.^17^ designed a cascaded chaotic mapping and proposed a secure image encryption scheme based on this mapping in 2007. The scheme uses the cascaded chaotic mapping as a pseudo-random number generator, which can achieve a very fast throughput. In 2008, Behnia et al.^18^ combined one-dimensional chaotic mapping with typical coupled map lattices to design a hybrid chaotic mapping that can achieve secure encryption within an acceptable speed range. In 2012, Wang et al.^19^ proposed an image encryption scheme based on iterative chaotic mapping, which is applicable to different types of images, has high randomness, and fast encryption speed. However, there are also some drawbacks, such as high computational complexity and potential performance impact on specific images. In 2014, Hussain et al.^20^ proposed an image encryption scheme based on S-box transformation and coupled map lattices, which verified that the S-box can meet 5 standards. In 2015, Tong et al.^21^ proposed an image encryption algorithm based on the Rabinyovich super-chaotic map, achieving high-dimensional chaotic encryption and improving security. In 2016, Hua et al.^22^ designed a two-dimensional logical adjustment sine map, which further extended the dynamic range of one-dimensional logical maps and sine maps and had a higher security level. In 2017, Pak et al.^23^ proposed a new chaotic map with a linear-nonlinear structure and proved its good chaotic characteristics. The main advantages of this scheme are enhanced encryption strength and security, and it can achieve higher encryption efficiency and compression of quantum images. Li et al.^24^ designed a quantum grayscale image encryption and compression scheme based on quantum cosine transform and five-dimensional hyperchaotic system. In order to improve the randomness of existing chaotic maps, Luo et al.^25^ proposed an image encryption scheme based on double chaotic systems in 2019, which uses a two-dimensional Baker map to control the state variables and system parameters of the logical map. In 2020, Jithin et al.^26^ proposed an image encryption scheme based on Arnold map, DNA encoding operation and Mandelbrot set to meet the requirements of efficient and secure encryption. In 2021, Zhang et al.^27^ proposed an image encryption scheme based on complex sine segmented linear chaotic mapping and variable DNA encoding. The main advantage of this scheme is the introduction of a composite chaotic system, which has superior dynamic performance and a larger parameter space, contributing to improved security and flexibility of the encryption algorithm. In 2022, Liu et al.^28^ designed a universal composite coupled chaotic model and demonstrated its feasibility. This model, with high dynamic complexity, can be applied to various scenarios. In 2023, Zhu et al.^29^ presented an image encryption scheme based on one-dimensional fractional-order sine mapping and parallel DNA encoding. This scheme addresses the drawbacks of common DNA-based image encryption algorithms and significantly improves the speed of encryption and decryption algorithms through parallel computation.

Most of the current quantum image encryption algorithms are designed for square images, and there is a lack of encryption algorithms suitable for non-square quantum images. Therefore, we propose a nonequal length image encryption algorithm using Arnold transformation and bit-plane chaotic mapping to address the aforementioned weaknesses. Prior to diffusion and confusion, the plain image undergoes bit-plane decomposition, resulting in two binary sequences of equal size. During the diffusion phase, a mutual diffusion strategy is employed between these sequences, effectively dispersing binary values. This ensures that even a small modification to the plain image leads to significant changes in the cipher sequences. In the confusion phase, binary elements are swapped between the two sequences using the control of the Lorenz chaotic system. This allows for the permutation of bits from one bitplane to any other, introducing additional complexity to the encryption process. Then, the Arnold algorithm is applied to scramble the ciphertext. This creates a cryptosystem that is highly resistant to differential attacks. Experimental and simulation results demonstrate the strong encryption performance of the proposed scheme. It provides effective encryption for non-square quantum images, addressing the weaknesses of existing algorithms in this aspect.

The remainder of this paper is organized as follows. Section "Binary bitplane decomposition and Lorenz chaotic map" provides a brief introduction to the basic theory of the GQIR image representation model, binary bit-plane decomposition, Lorenz chaos system, and Arnold algorithm. In Section "Proposed image encryption algorithm", we present a detailed description of the proposed algorithm. Section "Experimental results and performance analysis" offers simulation results and security analysis. Finally, the last section presents the conclusion of this paper.

## Binary bitplane decomposition and Lorenz chaotic map

### GQIR representation model

Nan Jiang et al.^11^ proposed a generalized quantum image representation for storing arbitrary integer quantum images. Theoretically, GQIR can flexibly store and extract the quantum image of size $$H \times W$$, where $$ X $$-axis ($$Y $$-axis) coordinate information is stored with $$w(h)$$ qubits, and the pixels of grayscale image information is stored with *q* qubits, which results in a total number of $$h + w + q$$ qubits. The plaintext quantum image $$\left| I \right\rangle$$ of size $$H \times W$$ in the GQIR is:

1 $$  \left| I \right\rangle  = \frac{1}{{\sqrt 2 ^{{h + w}} }}\sum\limits_{{Y = 0}}^{{H - 1}} {\sum\limits_{{X = 0}}^{{W - 1}} { \otimes _{{j = 0}}^{{q - 1}} \left| {C_{{YX}}^{j} } \right\rangle \left| {YX} \right\rangle } }  $$

where

2 $$  \begin{gathered}   h =  \left\{ {\begin{array}{*{20}l}    {\left\lceil {\log _{2} H} \right\rceil ,} \hfill & {H > 1} \hfill  \\    {1,} \hfill & {H = 1} \hfill  \\   \end{array} } \right. \hfill \\   w =  \left\{ {\begin{array}{*{20}l}    {\left\lceil {\log _{2} W} \right\rceil ,} \hfill & {W > 1} \hfill  \\    {1,} \hfill & {W = 1} \hfill  \\   \end{array} } \right. \hfill \\  \end{gathered}   $$

**Figure 1:**
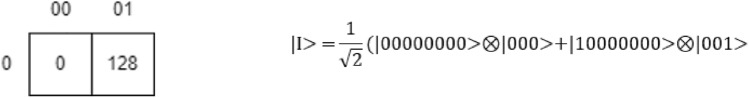
A example image and its representative expression in GQIR.

${C}_{YX}^{j}$ encodes the pixels of grayscale images information, $\left| {YX} \right\rangle$ represents the *X* coordinates $y_{0} y_{1} \ldots y_{\log 2 W - 1}$ and the *Y* coordinates $x_{0} x_{1} \ldots x_{\log 2 H - 1}$ in an image. Figure 1 shows a ${1} \times {2}$ grayscale image and its representative expression in GQIR.

### Binary bitplane decomposition

In Ref.^30^, Zhou presented three bitplane decomposition methods in detail. For our encryption algorithm, we have chosen to use the binary bitplane decomposition (BBD) method. In a grayscale image, each pixel value is represented by an 8-bit binary sequence with values ranging from 0 to 255. BBD can partition a grayscale image into 8 binary bitplanes, where each pixel's binary representation's *i*th bit is used to compose the ith bitplane.

A non-negative decimal number *N* can be represented by a binary sequence $(b_{{n - 1}} , \ldots ,b_{1} b_{0} )$ based on the following equation:

3 $$ N = \mathop \sum \limits_{i = 0}^{n - 1} b_{i} 2^{i} = b_{0} 2^{0} + b_{1} 2^{1} + \cdots + b_{n - 1} 2^{n - 1} $$

**Figure 2:**
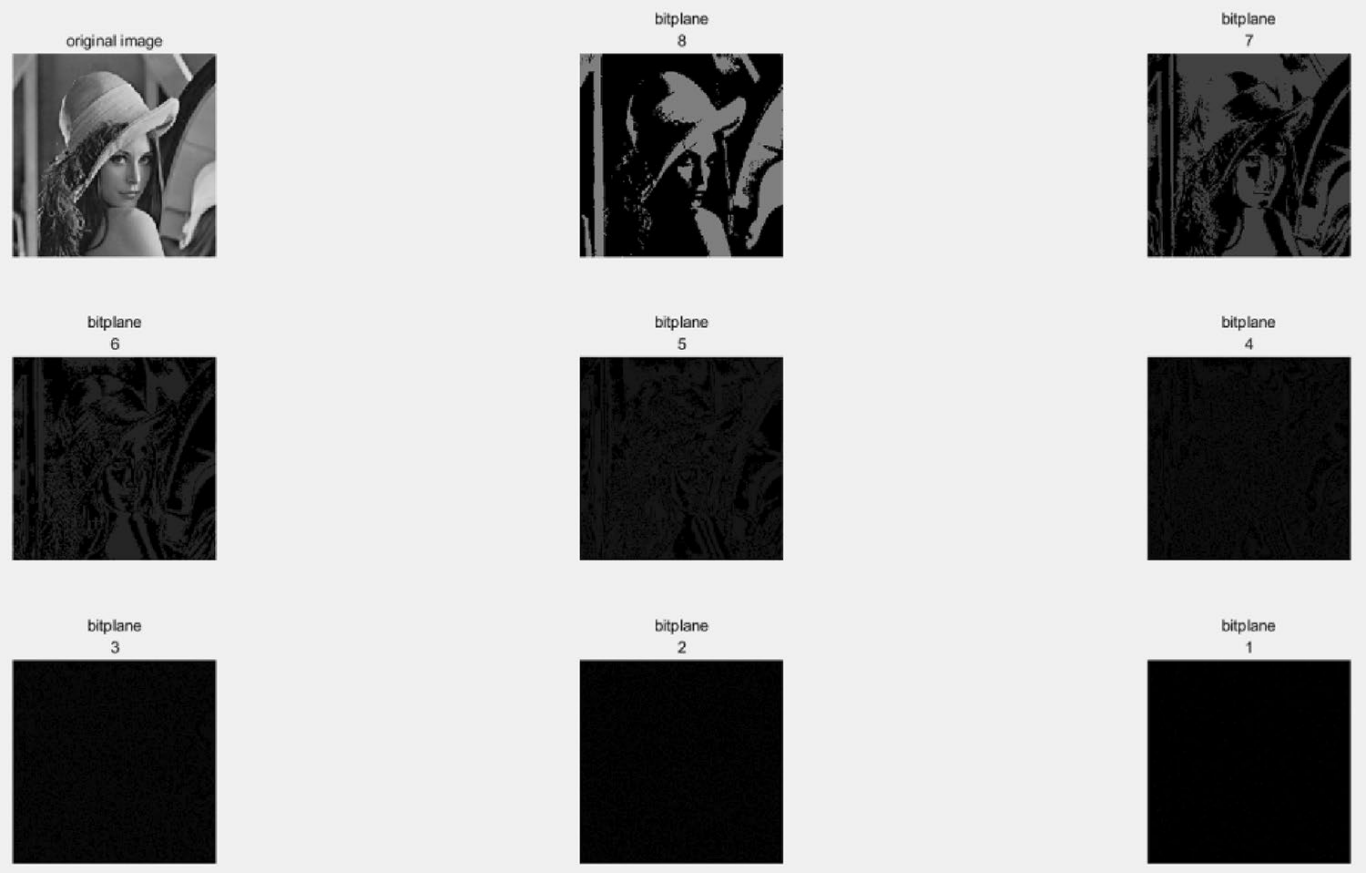
Image bitplane decomposition of the grayscale Lena image using BBD.

Among these bitplanes, higher bitplanes contain more significantly visual information of the original image while lower bitplanes show more details. Figure 2 shows the bitplane decomposition of the grayscale Lena image in Fig. 6a.

### The Lorenz chaotic map

During his study on convection experiments, Lorenz discovered a higher-dimensional dynamical system, which was one of the earliest continuous dynamical systems in the world to exhibit a singular attractor. It also displayed complex nonlinear dynamic behavior characteristics. The system describes the movements of fluid inside a heating barrel from the bottom of the bucket. This system is known as the Lorenz system^31^, the dynamic mechanics equations as follows:

4 $$  \left\{ {\begin{array}{*{20}l}    {\frac{{dx}}{{dt}} = a(y - x)} \hfill  \\    {\frac{{dy}}{{dt}} = bx - xz + cy} \hfill  \\    {\frac{{dz}}{{dt}} = xy - dz} \hfill  \\   \end{array} } \right.  $$

**Figure 3:**
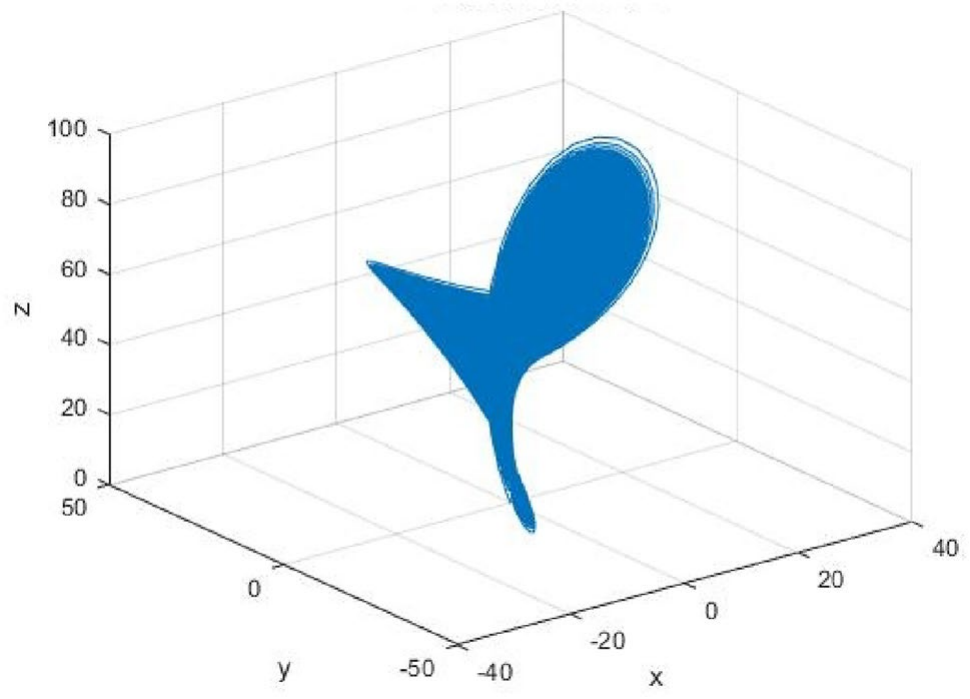
The projections of Lorenz attractor.

Among them: *a*, *b*, *c* and d for the Lorenz system parameters, are desirable any real number greater than zero, when taking $a = 10,\,b = 28,\,c = 1,\,d = 8/3$, Lorenz system exhibits a chaotic behavior, the projections of the chaotic attractor are shown in Fig. 3.

### Nonequal-length Arnold transformation

The nonequal-length Arnold transform is studied for carrier images of arbitrary size by the following Eq. (5):

5 $$ \left( {\begin{array}{*{20}c}    {x^{\prime } }  \\    {y^{\prime } }  \\   \end{array} } \right) = \left( {\begin{array}{*{20}c}    a & b  \\    c & d  \\   \end{array} } \right)\left( {\begin{array}{*{20}c}    x  \\    y  \\   \end{array} } \right)\bmod \left( {\begin{array}{*{20}c}    M  \\    N  \\   \end{array} } \right) $$

In order to ensure that Eq. (5) can be applied to image scrambling, the basic idea is to ensure that two points in the original carrier image cannot correspond to the same point after scrambling. In the literature^32–34^, the periodicity of the nonequal-length Arnold transform formula was investigated from the perspective of the length and width of the carrier image and the coefficient matrix, respectively. In order to ensure that the transformation formula is universal, i.e., it can be applied to carrier images of arbitrary size. We constrained the coefficient matrix by requiring $a = 1,b > 0,c = KN(\gcd (M,N))^{{ - 1}} ,d = 1 + bc$, where *b*, *k* are integers. According to the constraints of the coefficient matrix, Eq. (5) is equivalent to

6 $$  \left\{ {\begin{array}{*{20}l}    {x^{\prime }  = \left( {x + by} \right)\,\bmod \,M} \hfill  \\    {y^{\prime }  = [cx + (1 + bc)y]\,\bmod \,N} \hfill  \\   \end{array} } \right.  $$

**Figure 4:**
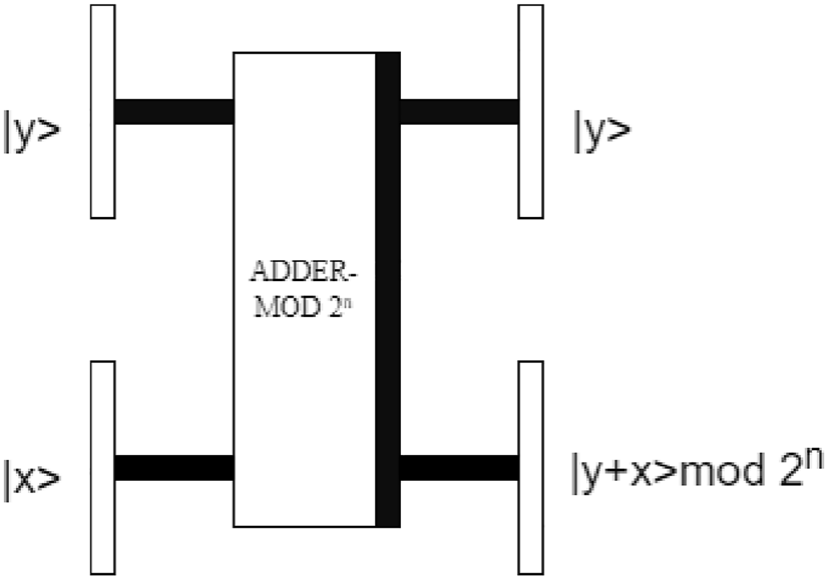
The quantum circuits for Arnold transform.

The classical Arnold transform is extended to the quantum version by Jiang et al.^35^, and the Arnold transform can be accomplished with quantum plain adder network and adder modulo *N* network. The corresponding quantum circuits for Arnold transform is shown in Fig. 4, and the detailed description can be found in^35^. The Arnold transform only changes the information of coordinates and the gray-scale information is remain unchanged. For a quantum image denoted as $\left| I \right\rangle$, one iteration of nonequal-length Arnold transform operation can be expressed as:

7 $$ \begin{aligned}   \left| {I^{\prime } } \right\rangle  =  & \psi \left( {\left| I \right\rangle } \right) = \frac{1}{{2^{n} }}\sum\limits_{{y{\text{ }} = {\text{ }}0}}^{{2^{n}  - 1}} {\sum\limits_{{x{\text{ }} = {\text{ }}0}}^{{2^{n}  - 1}} {\left| {C\left( {y,x} \right)} \right\rangle \psi \left( {\left| {yx} \right\rangle } \right)} }  \\       = & \frac{1}{{2^{n} }}\sum\limits_{{y{\text{ }} = {\text{ }}0}}^{{2^{n}  - 1}} {\sum\limits_{{x{\text{ }} = {\text{ }}0}}^{{2^{n}  - 1}} {\left| {C\left( {y,x} \right)} \right\rangle \psi \left( {\left| y \right\rangle } \right)\psi \left( {\left| x \right\rangle } \right)} }  \\  \end{aligned}  $$

where $\psi$ is the nonequal-length Arnold transform operation. $\left| {I^{\prime } } \right\rangle$ is the scrambled image.

Similar to the classical Arnold transform, the scrambled coordinates of quantum image $\left| I \right\rangle$ can be written as:

8 $$  \left\{ {\begin{array}{*{20}l}    {\left| {x^{\prime } } \right\rangle  = \left| {x + by} \right\rangle \bmod \,M} \hfill  \\    {\left| {y^{\prime } } \right\rangle  = \left| {cx + (1 + bc)y} \right\rangle \bmod \,N} \hfill  \\   \end{array} } \right.  $$

Based on Eq. (8), the inverse nonequal-length Arnold transform can be easily derived as follows:

9 $$   \left\{ {\begin{array}{*{20}l}    {\left| y \right\rangle  = \left| {y^{\prime }  - cx^{\prime } } \right\rangle \bmod \,N} \hfill  \\    {\left| x \right\rangle  = \left| {x^{\prime }  - by} \right\rangle \bmod \,M} \hfill  \\   \end{array} } \right.   $$

## Proposed image encryption algorithm

**Figure 5:**
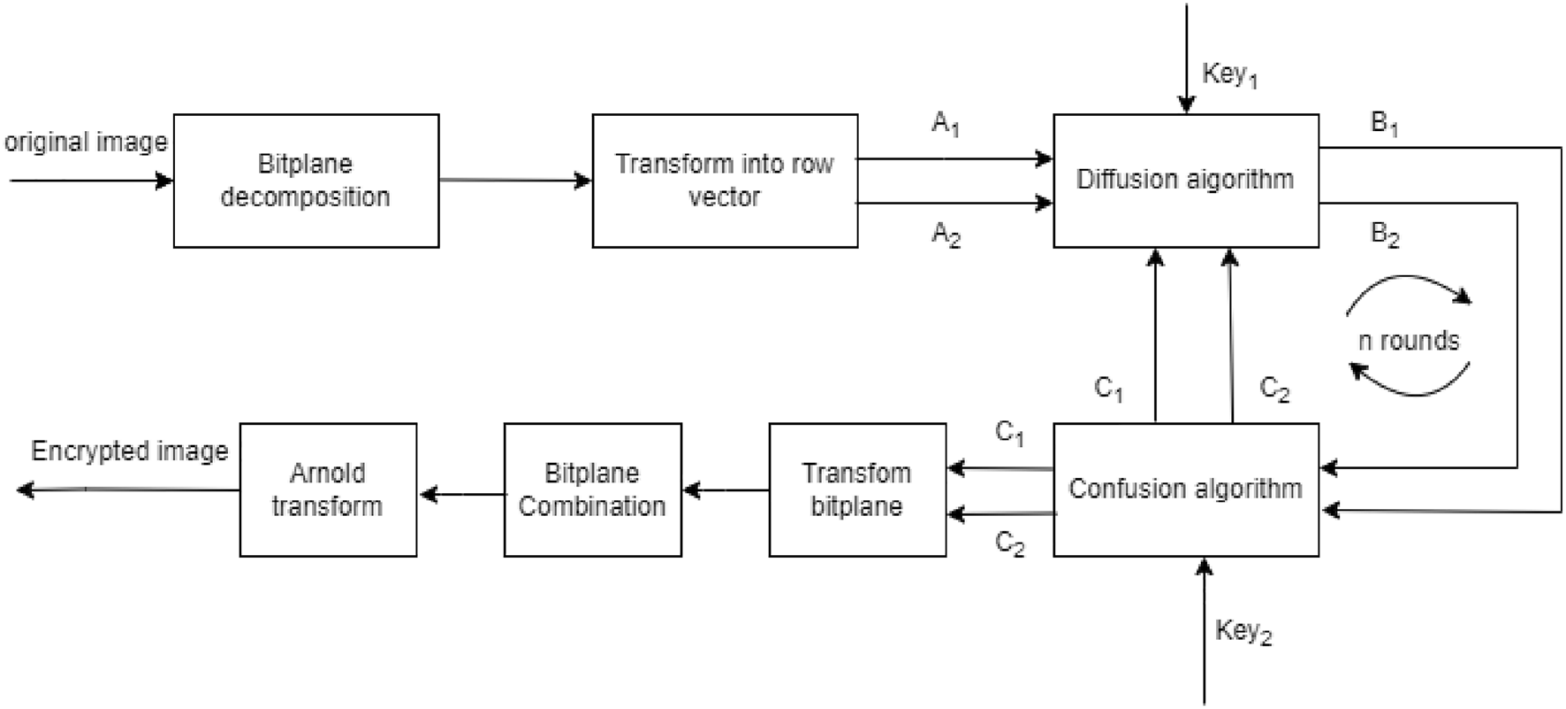
Block diagram of the proposed image cryptosystem.

The proposed image encryption algorithm is illustrated in Fig. 5. Initially, the plain image is decomposed into eight bitplanes $(i_{1} ,i_{2} , \ldots ,i_{8} )$ using the Bit-Plane Decomposition (BBD) technique.

These bitplanes are divided into two groups, $A_{{a1}}$ and $A_{{a2}}$, with an equal number of bitplanes in each group. For example, we can choose four higher bitplanes for one group and four lower bitplanes for the other group.

Then, we transform these two groups into two binary sequences, $A_{1}$ and $A_{2}$. The elements of the bitplanes are arranged sequentially from top to bottom, left to right, and from higher to lower bitplanes, forming the sequences $A_{1}$ and $A_{2}$.

Before proceeding to the confusion and diffusion phase, we generate two binary keystream sequences using a secret key, represented as $key_{1} \left( {x_{0} ,y_{0} ,z_{0} ,a,b,c,d} \right)$. Assuming that the size of the plain image is $M \times N$, we set the initial parameters $a,b,c,d$, and the initial values $x_{0} ,y_{0} ,z_{0}$ to iterate the Lorenz map (Eq. 4) $N_{0}  + MN$ times. We discard the initial $N_{0}$ values to avoid any potential adverse effects. The resulting chaotic sequence has *MN* elements denoted as $X = \left\{ {x_{1} ,x_{2} , \ldots x_{{MN}} } \right\}$. To convert $X\left( i \right)$ into an integer sequence $X_{1} \left( i \right)$, we employ the following formula.

10 $$ X_{{1}} {\text{ = mod}}\left( {{\text{floor}}\left( {X \times {10}^{{{14}}} } \right){,256}} \right) $$

The elements in $X_{1} \left( i \right)$ are within the range of 0 to 255. We utilize the BBD technique to decompose *X* into eight bitplanes, resulting in eight binary sequences. These sequences can be flexibly divided into two groups of equal size. For instance, we can choose four odd bitplanes for one group and four even bitplanes for the other group.

To generate the binary keystream sequences, we transform these two groups into two binary sequences, denoted $b_{1}$ and $b_{2}$, respectively. The elements of the bitplanes are arranged sequentially from left to right and from higher bitplanes to lower bitplanes, forming the sequences $b_{1}$ and $b_{2}$.

Below, the confusion and diffusion phases are described in detail.

### Diffusion phase

The detailed steps of the diffusion phase are as follows:

- Step 1. Obtain parameter $sum_{1}$. To calculate the sum of the elements in $A_{2}$, we can use Eq. (11) as follows:
  11 $$  sum_{1}  = \sum\limits_{{i = 1}}^{L} {A_{2} } \left( i \right)  $$
  where $L$ is the size of $A_{1}$ and $A_{2}$, and $L = 4MN$.
- Step 2. Obtain matrix $$ A_{{11}}  $$. We need to perform a cycle shift operation on the binary matrix $$ A_{1}  $$. This operation shifts the elements of $$ A_{1}  $$ to the right by $$ sum_{1} $$ bits, resulting in the cyclic shift matrix $$ A_{{11}}$$.
- Step 3. Encrypt the first element of matrix $$ A_{{11}}$$. After obtaining $$ A_{{11}}$$, we can proceed to encrypt its first element. To do this, we use the last element in $$ A_{{11}}$$, the first element in $$ A_{2} $$ and $$ b_{1}  $$, according to Eq. (12).
  12 $$  B_{1} \left( 1 \right) = A_{{11}} \left( 1 \right) \oplus A_{{11}} \left( L \right) \oplus A_{2} \left( 1 \right) \oplus b_{1} \left( 1 \right)  $$
  By performing the bit-level XOR operation between these elements and applying the formula, we can obtain the encrypted value of the first element in $$ A_{{11}}$$, denoted as $$ A_{{11}}$$ (1,1).
- Step 4. Encrypt the remaining elements of matrix $$ A_{{11}}$$. After encrypting the first element in $$ A_{{11}}$$ in step 3 of the algorithm, we set $$ i = 2  $$ and proceed to encrypt the *i*th element in $$ A_{{11}}$$ using the ith element in $$ A_{2} $$ and $$ b_{1}  $$. This operation can be performed using Eq. (13), which is given as:
  13 $$  B_{1} \left( i \right) = A_{{11}} \left( i \right) \oplus A_{{11}} \left( {i - 1} \right) \oplus A_{2} \left( i \right) \oplus b_{1} \left( i \right)  $$
  Here, $$  A_{{11}} (n,\,i - 1) $$ denotes the $$ (i - 1) $$ th element in the last row of $$ A_{{11}}$$, $$ A_{2} $$ $$ (1,i) $$ represents the *i*th element in $$ A_{2} $$ and $$ b_{1}  ({\text{i}})$$ denotes the *i*th element in the binary sequence $$ b_{1}  $$.The bit-level XOR operation is performed between these elements and the formula is applied.
- Step 5. Cycle XOR operation. Set $i = i + 1$, and return to step 4 until *i* reaches *L*. Use the same method to encrypt $A_{2}$.
- Step 6. Obtain parameter $$ sum_{2} $$. Calculate the sum of the elements in $$ B_{1}$$ according to Eq. (14).
  14 $$  sum_{2}  = \sum\limits_{{i = 1}}^{L} {B_{1} } \left( i \right) $$
- Step 7. Obtain matrix $$ A_{{22}}$$. We need to perform a cycle shift operation on the binary matrix $$ A_{2} $$. This operation shifts the elements of $$ A_{2} $$ to the right by $$ sum_{2} $$ bits, resulting in the cyclic shift matrix $$ A_{{11}}$$.
- Step 8. Encrypt the first element of matrix $$ A_{{22}}$$. After obtaining $$ A_{{22}}$$, we can proceed to encrypt its first element. To do this, we use the last element in $$ A_{{22}}$$, and the first elements in $$ B_{1}$$ and $$ b_{2}  $$, according to Eq. (15).
  15 $$ B_{2} \left( 1 \right) = A_{22} \left( 1 \right) \oplus A_{22} \left( L \right) \oplus B_{1} \left( 1 \right) \oplus b_{2} \left( 1 \right) $$
  By performing the bit-level XOR operation between these elements and applying the formula, we can obtain the encrypted value of the first element in $$ A_{{22}}$$, denoted as $$ A_{{22}}$$ (1,1).
- Step 9. Encrypt the remaining elements of matrix $$ A_{{22}}$$. After encrypting the first element in $$ A_{{22}}$$ in step 8 of the algorithm, we set $$ i = 2 $$ and proceed to encrypt the *i*th element in $$ A_{{22}}$$ using the $$i$$ th element in $$ B_{1}$$ and $$ b_{2}  $$. This operation can be performed using Eq. (16), which is given as:
  16 $$ B_{2} \left( i \right) = A_{22} \left( i \right) \oplus A_{22} \left( {i - 1} \right) \oplus B_{1} \left( i \right) \oplus b_{2} \left( i \right) $$
  Here, $$  A_{{22}} (n,i - 1) $$ denotes the $$ (i - 1) $$ th element in the last row of $$ A_{{22}}$$, $$ B_{1}$$ $$ (1,i) $$ represents the $$i$$ th element in $$ B_{1}$$ and $$  b_{2} (i) $$ denotes the $$i$$ th element in the binary sequence $$ b_{2}  $$. The bit-level XOR operation is performed between these elements and the formula is applied.
- Step 10. Cycle XOR operation. Set $i = i + 1$, and return to step 9 until *i* reaches *L*.

### Confusion phase

The detailed steps of the confusion phase are as follows:

- Step 1. Sequences *Y* and *Z* are generated using the Lorenz chaotic system. The secret key $key_{2} \left( {x_{0}^{\prime } ,y_{0}^{\prime } ,z_{0}^{\prime } ,a^{\prime } ,b^{\prime } ,c^{\prime } ,d^{\prime } } \right)$ is used to produce the chaotic sequences *Y* and *Z*. The initial value $s_{0}$ is set according to Eq. (17).
  17 $$ s_{0}  = \bmod \left( {\frac{{sum_{2} }}{L},1} \right) $$
  To ensure the security of the encryption algorithm, it is necessary to iterate the chaotic system $N_{0}  + 2L$ times and discard the former $N_{0}$ values to avoid any potential harmful effects. The resulting chaotic sequence has 2 $L$ elements $S = \left\{ {s_{1} ,s_{2} , \ldots , s_{2L} } \right\}$.
  Next, we divide the sequence *S* into two equal parts using Eqs. (18, 19).
  18 $$ S_{1} = \left\{ {s_{1} , s_{2} , \ldots ,s_{L} } \right\} $$
  19 $$ S_{2} = \left\{ {s_{L + 1} , s_{L + 2} , \ldots ,s_{2L} } \right\} $$
  To convert the chaotic sequences $S_{1}$ and $S_{2}$ to integer sequences *Y* and *Z*, each with a length of *L*, we can use the following formula:
  20 $$ Y = \bmod \left( {{\text{floor}}\left( {S_{1} \times 10^{14} } \right),L} \right) + 1 $$
  21 $$ Z = \bmod \left( {{\text{floor}}\left( {S_{2} \times 10^{14} } \right),L} \right) + 1 $$
- Step 2. Exchange elements of $$ B_{1}$$ and $$ B_{2}$$ by the sequence *Y*. Set $$ i{\text{ }} = 1 $$, and swap the binary elements in $$ B_{1}  $$ and $$ B_{2}  $$ according to Eqs. (22–24).
  22 $$ temp = B_{1} \left( i \right) $$
  23 $$ B_{1} \left( i \right) = B_{2} \left( {Y\left( i \right)} \right) $$
  24 $$ B_{2} \left( {Y\left( i \right)} \right) = temp $$
- Step 3. Cycle swap operation. Set $i = i + 1$; and return to step 2 until *i* reaches *L*.
- Step 4. Exchange elements of $$ B_{1}$$ and $$ B_{2}$$ by the sequence *Z*. Set $$ j = 1 $$; and swap the binary elements in $$ B_{1}$$ and $$ B_{2}$$ according to Eqs. (25–27).
  25 $$ temp = B_{2} \left( j \right) $$
  26 $$  B_{2} \left( j \right) = B_{1} \left( {Z\left( j \right)} \right) $$
  27 $$  B_{1} \left( {Z\left( j \right)} \right) = temp $$
- Step 5. Cycle swap operation. Set $j = j + 1$; and return to step 4 until *j* reaches *L*.
  Then, we obtain the encrypted row vectors $C_{1}$ and $C_{2}$. We transform the sequences $C_{1}$ and $C_{2}$ into an $M \times N$ image *C*.

### Nonequal-length Arnold transformation

We need to process the quantum bits that represent the position information in the GQIR model of image *C*. According to Eq. (8), the coordinates of the encrypted image are defined as follows:

28 $$  \left\{ {\begin{array}{*{20}l}    {x^{\prime }  = \left( {x + by} \right)\bmod M} \hfill  \\    {y^{\prime }  = (cx + \left( {1 + bc} \right)y)\bmod N} \hfill  \\   \end{array} } \right.  $$

${x^{\prime } }$ and ${y^{\prime } }$ are the coordinate information of the final quantum encrypted image *E*, *x* and *y* are the coordinate information of quantum image *C*, and *M* and *N* denote the length and width of the image, respectively.

### Quantum image decryption system

As the quantum operations are invertible, the decryption process is exactly the inverse process of encryption. The image before the Arnold transformation can be recovered by performing inverse nonequal-length Arnold transformation on quantum image *E* according to the parameters used in the encryption. According to Eq. (9), the coordinates of the decrypted image are defined as follows:

29 $$ \left\{ {\begin{array}{*{20}c}    {y = (y^{\prime }  - cx^{\prime } )\bmod N}  \\    {x = (x^{\prime }  - by)\bmod M}  \\   \end{array} } \right. $$

Then the inverse operations of the confusion and diffusion phases are performed sequentially to obtain the original image. The decryption procedure is the reverse process of encryption, but focused attention must be given to the reverse order of the cyclic shift and swap.

## Experimental results and performance analysis

Due to the lack of practical quantum computers in reality, our experimental results were simulated using a classical computer equipped with the MATLAB environment. MATLAB is an excellent tool that facilitates the representation and manipulation of large vectors and matrix arrays, allowing us to effectively simulate quantum states and operators, such as superposition states of quantum images and quantum unitary operations.

**Figure 6:**
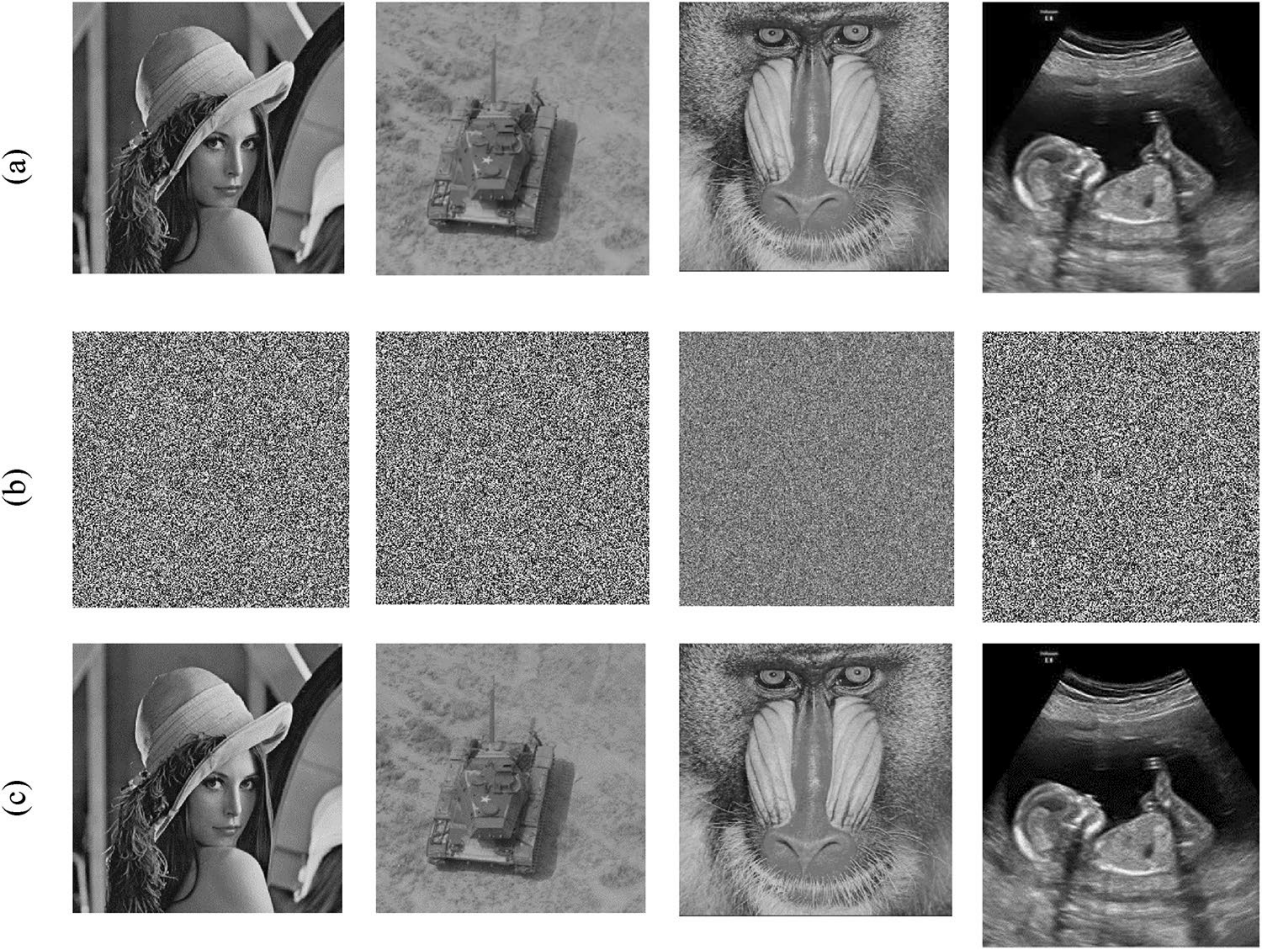
(**a**) Tested images used in our simulation. (**b**) Visual effects of the encrypted grayscale images. (**c**) Visual effects of the decrypted grayscale images.

To evaluate the performance of our presented quantum encryption scheme, four grayscale images (Lena, Tank, Baboon, and Child) are used as test images, and their sizes are 256*256, 256*256, 512*512, and 234*246, respectively, as shown in Fig. 6a. The corresponding encrypted images and decrypted images are shown in Fig. 6b and c.

### Security key space

Whether the key space of an algorithm is large enough to resist exhaustive attack by an attacker is an important aspect of judging the merit of an encryption algorithm. In the proposed algorithm, the secret keys include the control parameter and the initial values of two Lorenz chaotic systems $\left( {x_{0} ,y_{0} ,z_{0} ,x_{0}^{\prime } ,y_{0}^{\prime } ,z_{0}^{\prime } } \right)$; round n; iteration times $N_{0}$; and positive integer *k*, which denote the iteration times of the Arnold transform implemented on the plain image pixel coordinates. If the double precision type of data is 64 bits in length, then the key space of the proposed encryption algorithm is $k \times n \times N_{0}  \times \left( {2^{{64}}  \times 2^{{64}}  \times 2^{{64}} } \right)^{2}  \to \infty$. If the parameters *a*, *b*, *c* and *d* of the Lorenz chaos system are also used as key parameters, then the key space is larger. The total key space is significantly larger than that in^36,37^, whose key space are ${2}^{210}$ and ${10}^{48}$,respectively. Therefore, the key space of the proposed encryption algorithm is large enough to effectively prevent exhaustive attacks.

### Key sensitivity analysis

**Figure 7:**
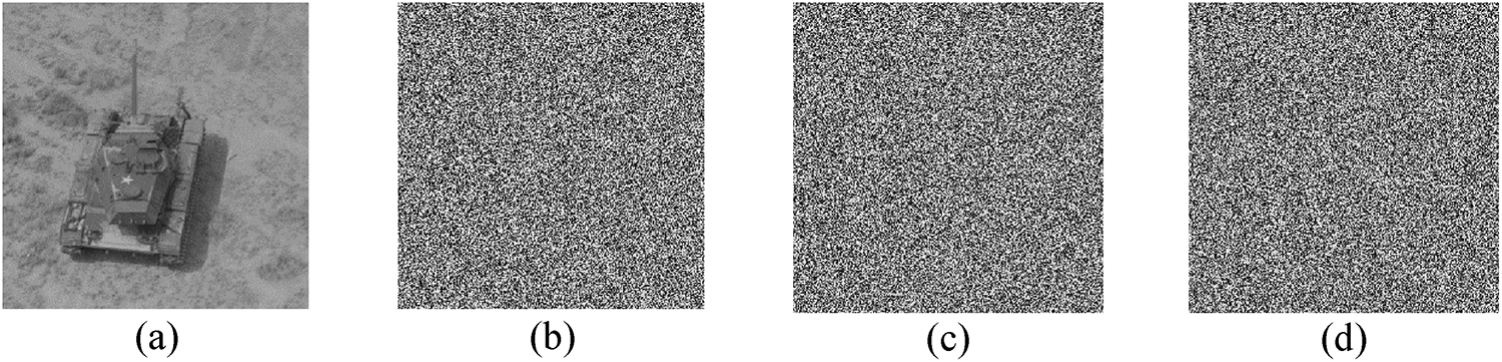
Key space of the Lorenz chaotic system based scheme.

Key sensitivity is an important indicator of the security of an encryption algorithm; the higher the key sensitivity is, the greater the security of the encryption algorithm. Key sensitivity means that a very small change in the key can lead to a failure in decryption. Here, we use the chaotic sequence encryption algorithm, and the encryption and decryption keys are the initial values of the chaotic system. We take the key of the Lorenz chaotic system as an example; its key is the initial value of the system $\left( {x_{0} ,y_{0} ,z_{0} ,x_{0}^{\prime } ,y_{0}^{\prime } ,z_{0}^{\prime } } \right)$, and the initial value of the encryption sequence used in Fig. 7a is (0.001,0.005,0.002). To verify the key sensitivity of this algorithm, only the initial value $x_{0} ,y_{0} ,z_{0}$ is used. Make a very slight change to change their value to $x_{0}  + 10^{{ - 15}} ,y_{0}  + 10^{{ - 15}} ,z_{0}  + 10^{{ - 15}}$ separately, that is, the keys become ($0.001 + 10^{{ - 15}}$, 0.005,0.002), ($0.001$, 0.005 $+ 10^{{ - 15}}$,0.002) and ($0.001$, 0.005,0.002 $+ 10^{{ - 15}}$), decrypt the encryption result with the correct key, and decrypt the encryption result with the changed key. The obtained results are shown in Fig. 7b–d. The above analysis reveals that the keys in the proposed scheme are sensitive.

### Histogram analysis

**Figure 8:**
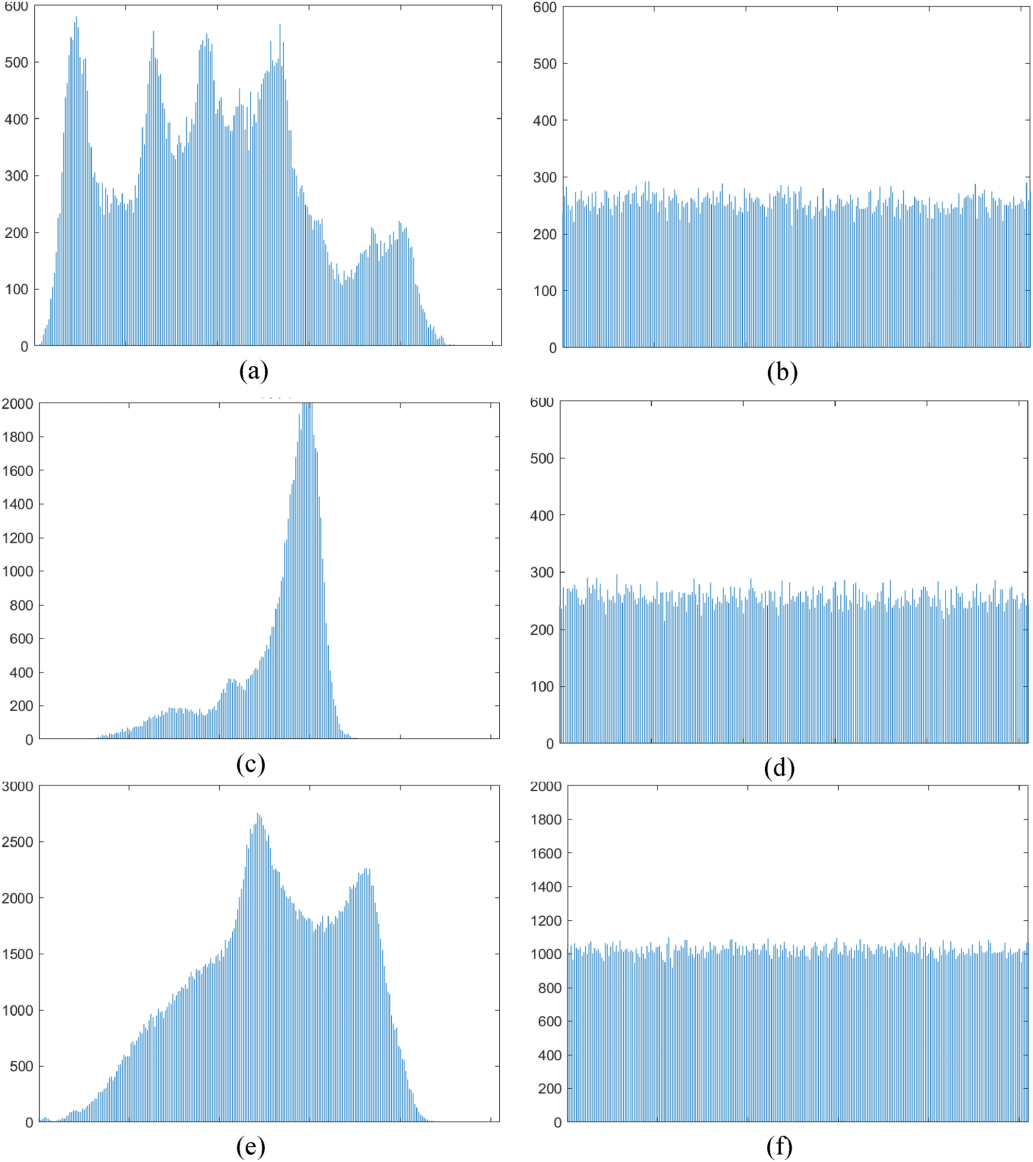
(**a**, **c** and **e**) are the histograms of the original images Lena, Tank and Baboon respectively, (**b**, **d** and **f**) are the histograms of the encrypted images Lena, Tank and cameraman respectively.

An image histogram reflects the distribution of an image’s pixel gray value, which is an essential metric for assessing the performance of any image encryption algorithm. A good secure encryption algorithm should guarantee that the histograms of encrypted images are completely different from the histograms of the original versions. Figure 8a,c and e shows the histograms of the original plain images. Figure 8b,d and f illustrates the histogram of encrypted images. Obviously, the histograms of the encrypted grayscale images from Lena, Tank and Baboon are completely different from those of the original versions. Therefore, we can conclude that there is no similarity in terms of histograms between the plain images and the encrypted versions.

### Correlation analysis

**Figure 9:**
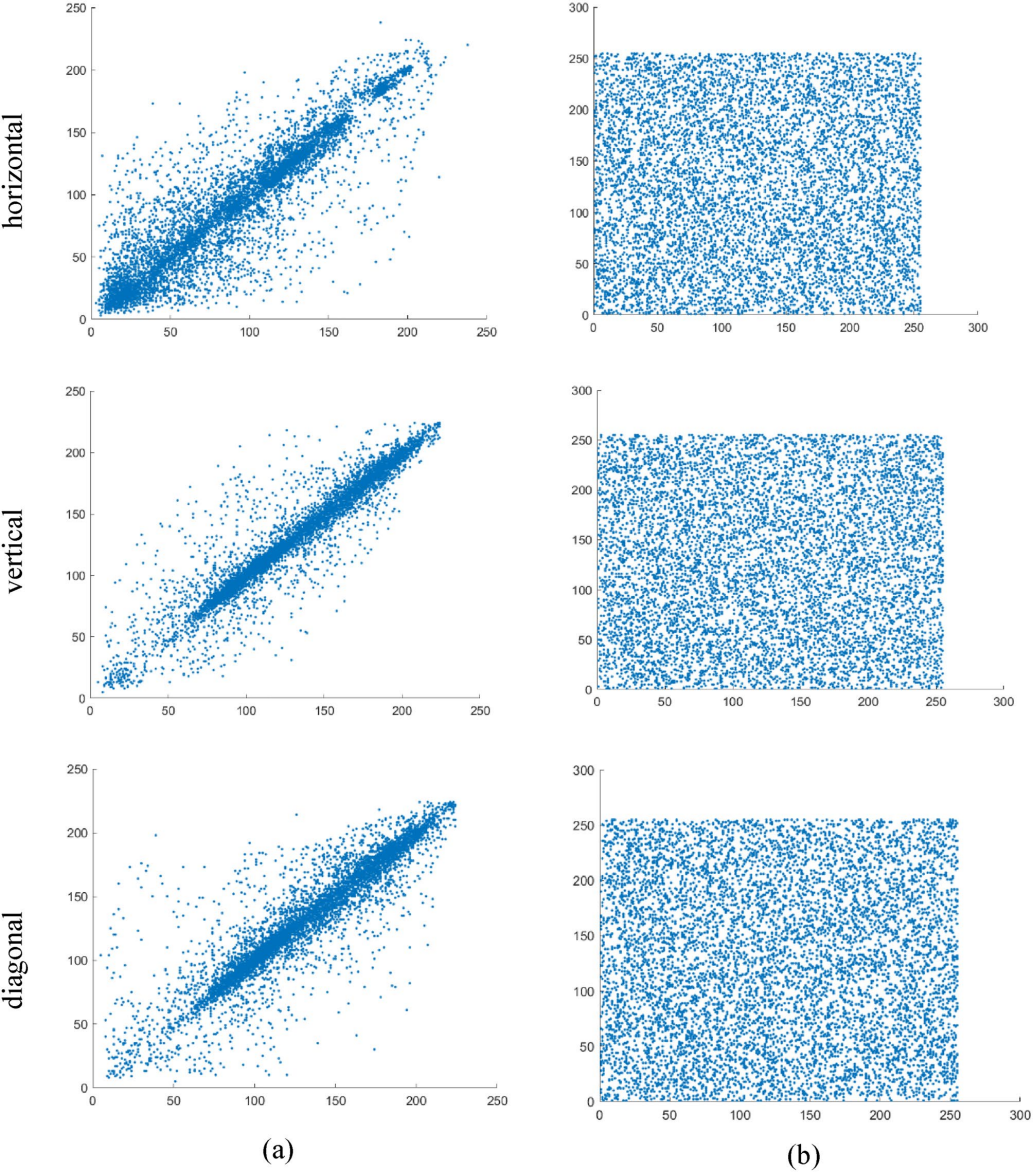
Correlation distributions between two adjacent pixels in three directions: (**a**) image Lena and (**b**) encrypted image Lena.

**Figure 10:**
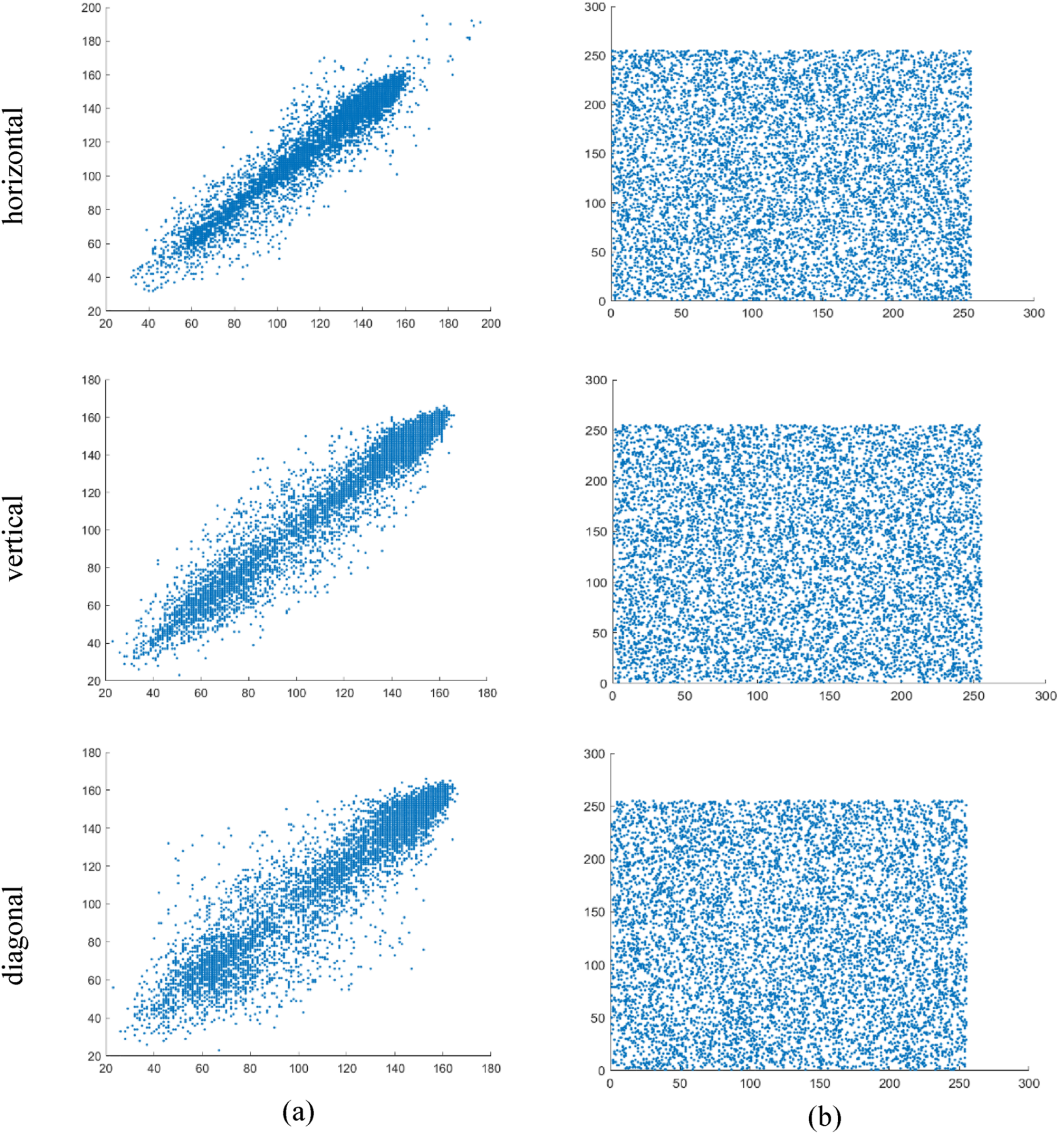
Correlation distributions between two adjacent pixels in three directions: (**a**) image Tank and (**b**) encrypted image Tank.

The correlation coefficient is an index that measures the degree of linear correlation between two random variables. Its value is located in the interval [− 1,1], and the absolute value of the correlation coefficient indicates the degree of correlation between variables. The diffusion process proposed in this chapter causes the position and pixel value of the plaintext image to change greatly. The correlation distributions of two adjacent pixels in three directions are shown in Figs. 9 and 10. As shown in the figure, the pixel values of the plaintext image are strongly correlated in all directions, while the pixel values of the ciphertext image are not correlated in all directions, and are evenly distributed in the two-dimensional region ranging from 0 to 255. Moreover, the correlation coefficients of 8000 adjacent pixel pairs in different directions were calculated according to Eq. (33). The comparison of the proposed encryption scheme with the correlation coefficients from other literature is listed in Table 1. From the table, it can be observed that the correlation coefficients of adjacent pixels in the plaintext and ciphertext images are close to 1 and 0, respectively, indicating that the encryption scheme significantly reduces the correlation of adjacent pixels in the plaintext image. Therefore, the quantum image encryption algorithm proposed in this chapter demonstrates strong resistance against statistical analysis.

**Table 1:** CC values of plaintext and ciphertext images in three directions.

Algorithm	Image	Horizontal direction	Vertical direction	Diagonal direction
	Lena	0.9069	0.9480	0.9226
Proposed scheme	Ciphertext image	0.0073	0.0115	− 0.0012
Ref.^38^		− 0.0368	0.0457	− 0.0076
Ref.^39^		0.0095	0.0202	− 0.0097
	Baboon	0.9333	0.8457	0.8121
Proposed scheme	Ciphertext image	− 0.0086	0.0056	0.0092
Ref.^40^		0.0063	0.0156	0.0072
Ref.^41^		0.0096	− 0.0110	0.0005

Additionally, we utilized the following formulas to calculate the correlation coefficient $r_{{xy}}$ for each pair:

30 $$  r_{{xy}}  = \text{cov} \left( {x,y} \right)/\sqrt {D\left( x \right)D(y)}  $$

31 $$ E\left( x \right) = \frac{1}{S}\sum\limits_{{i = 1}}^{S} {x_{i} }  $$

32 $$ D(x) = \frac{1}{S}\sum\limits_{{i = 1}}^{S} {(x_{i}  - E(x))^{2} }  $$

33 $$ \text{cov} \left( {x,y} \right) = \frac{1}{S}\sum\limits_{{i = 1}}^{S} {(x_{i}  - E(x))(y_{i}  - E(y))}  $$

where *x* and *y* represent the grayscale values of two neighboring pixels in the image, and *S* corresponds to the total number of pixels chosen from the image.

### Information entropy analysis

The statistical measure of the distribution of pixels in each layer of an image is called the information entropy. The information entropy of an image can be calculated using Eq. (34).

34 $$ H(m) = \mathop \sum \limits_{i = 0}^{{2^{n} - 1}} p(m_{i} )\log_{2} \frac{1}{{p(m_{i} )}} $$

where *n* represents the number of bits required to represent the symbol $m_{i}$, and $p(m_{i} )$ denotes the probability of symbol *m*. As observed in Eq. (34), the maximum entropy of an 8-bit grayscale image is 8 when all the pixels are equally distributed, indicating a random distribution of information. Therefore, after encryption, the information entropy of the encrypted image should approach 8. The closer the number of nodes is to 8, the less feasible it becomes for attackers to decrypt the cipher image. To calculate the information entropy of both the plain and cipher images, we employ Eq. (34). The results are presented in Table 2. Compared to other algorithms^42–44^, our encryption algorithm’s entropy is quite near the ideal value and can effectively resist an entropy attack.

**Table 2:** Information entropy of the original and encrypted images (bit).

Images	Information entropy	
	Original	Ciphertext
Lena	7.5683	7.9897
Tank	6.2779	7.9902
Baboon	7.3577	7.9915
Child	6.6876	7.9617
Lena^42^		7.9812
Lena^43^		7.9627
Lena^44^		7.9117

### Robustness analysis

The concept of robustness in image encryption refers to the strong ability to resist attacks. During the image encryption process, unexpected scenarios such as cropping, translation, compression, and noise interference may occur. In such cases, the decrypted image should maintain a high level of fidelity, at the very least being able to reproduce the original image. This algorithm employs unequal amounts of noise and partial removal attacks.

#### Noise attack analysis

During the transmission process, the encrypted image is usually influenced by noise. In this subsection, to test the robustness of resisting noise attacks, salt & pepper noise is added to the encrypted image.

**Figure 11:**
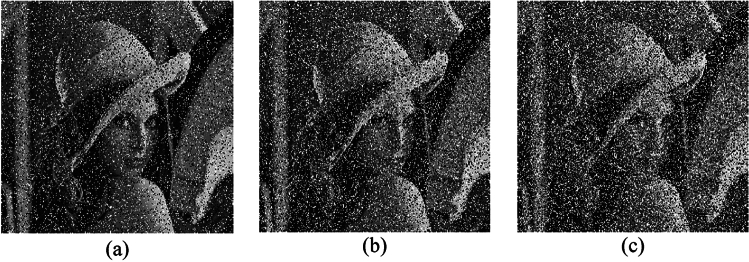
Decrypted images with different noise density: (**a**) noise density is 0.1, (**b**) noise density is 0.2, and (**c**) noise density is 0.3

Taking the encrypted Lena image shown in Fig. 6b as an example, the corresponding decrypted images when noise density is 0.1, 0.2, and 0.3 are shown in Fig. 11. As noise density increases, the decrypted images become increasingly blurred, but the main information can still be identified. Therefore, the proposed encryption scheme can resist noise attacks to a certain extent.

#### Cutting attack analysis

**Figure 12:**
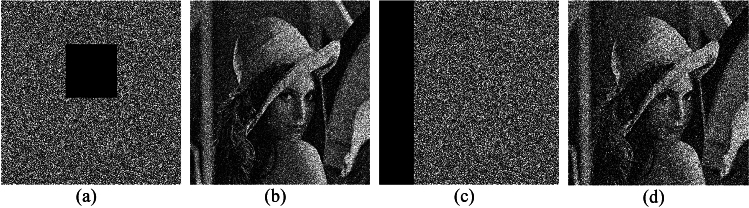
Sheared images in different positions and the corresponding decryption images.

To assess the capability of the encryption scheme to recover plaintext images from partially lost ciphertext data, i.e., its resilience against clipping attacks, we deliberately removed portions of the encrypted image and subsequently restored the original information from the remaining content. Figure 12 visually presents the decrypted images under various occlusion scenarios, revealing that a significant portion of the original information can be successfully reconstructed. Consequently, the proposed scheme exhibits a certain degree of resistance against occlusion attacks.

### Mean square error

A perfect encrypted image should significantly differ from the original image. The mean square error (MSE) is an effective metric that characterizes the difference between encrypted images and original versions. For two grayscale images with a size of $M \times N$, the MSE is defined as:

35 $$ {\text{MSE}} = \frac{1}{M \times N}\mathop \sum \limits_{i = 0}^{M} \mathop \sum \limits_{j = 0}^{N} \left[ {I\left( {i,j} \right) - E(i,j)} \right]^{2} $$

where $I(i,j)$ and $E(i,j)$ are the pixel gray values of the original and encrypted images respectively, at position $(i,j)$.

Obviously, the larger the MSE value is, the better the encryption effect. Table 3 presents the MSE values of the encrypted images in our proposal as well as those of^45,46^. The MSE of our proposed scheme is higher than that of their proposed scheme.

**Table 3:** MSE values of the encrypted color images with the original version.

MSEs Images	MSE values of the encrypted grayscale images with original version
Lena	9.0423e + 03
Tank	9.0431e + 03
Baboon	7.2615e + 03
Child	1.2145e + 04
Lena^45^	4.3110e + 03
Lena^46^	9.0052e + 03

### Computational and complexity analysis

The proposed quantum image encryption scheme consists of three main processes, namely diffusion, confusion, and Arnold scrambling. Therefore, the computational complexity primarily depends on the operations of diffusion, confusion, and Arnold scrambling. The complexity of quantum algorithms is typically measured by the number of logic gates involved. In the diffusion phase, the time-consuming part involves *O*(4*MN*) swap operations. To analyze the time complexity of confusion, the computational cost includes *O*(3*MN*) floating-point operations for constructing chaotic sequences in the Lorenz system, as well as *O*(4*MN*) shift and XOR operations. Since the Arnold transform implemented on pixel coordinates and color information is independent (i.e., these two processes can be executed in parallel), the time complexity for the two consecutive scramblings in this phase is *O*($\log _{2} MN$). Therefore, the overall time complexity is *O*(*MN*). Compared to Xu's algorithm^36^, the proposed scheme achieves faster speed as Xu's algorithm requires additional loop addition and modulo operations.

The implementation environment of the proposed algorithm is Matlab (R2020b). The encryption algorithm was tested on a personal computer with an Intel Core 1.80 GHz CPU and 4 GB memory. The average encryption speed is 2.06 s, while the encryption times for other schemes^47,48^ are 2.44 s and 2.135 s, respectively. Comparing the results shows that the proposed algorithm has stronger efficiency and is more suitable for real-time applications.

### Differential attack

To examine the impact of a single pixel variation on the overall encryption output of the algorithm, two commonly used measures, namely the Normalized Pixel Change Rate (NPCR) and the Unified Average Changed Intensity (UACI), were employed. NPCR quantifies the rate of change in the encrypted image when only one pixel in the source image is altered. UACI is utilized to gauge the average intensity of alterations between the source and encrypted images. The formulas for calculating NPCR and UACI are given in Eqs. (36) and (37) respectively.

36 $$  {\text{NPCR}} = \frac{{\sum\nolimits_{{i,j}} D (i,j)}}{{M \times N}} \times 100\%   $$

37 $$ UACI = \frac{1}{M \times N}\left[ {\mathop \sum \limits_{i,j} \frac{{\left| {C_{1} \left( {i,j} \right) - C_{2} \left( {i,j} \right)} \right|}}{255}} \right] \times 100\% $$

where *M* and *N* represent the width and height of either $C_{1}$ or $C_{2}$. For an ideal encryption algorithm, the calculated NPCR and UACI values should fluctuate around 99.6% and 33.4%, respectively.

We randomly altered the pixel values of grayscale images "Lena," "Tank," and "Baboon" and performed NPCR and UACI tests. From the Table 4, it can be observed that the achieved values closely match the theoretical values of UACI at 33.4635% and NPCR at 99.6094%^49^. Significant changes are evident in the encrypted image compared to the algorithm described in^50^. This demonstrates the strong resistance capability of our algorithm against differential attacks.

**Table 4:** NPCR and UACI results.

Images	NPCR (%)	UACI (%)
Lena	99.6093	33.4635
Lena^50^	99.58	34.08
Baboon	99.8046	33.3984
Baboon^50^	99.58	27.31

### Spectral analysis

**Figure 13:**
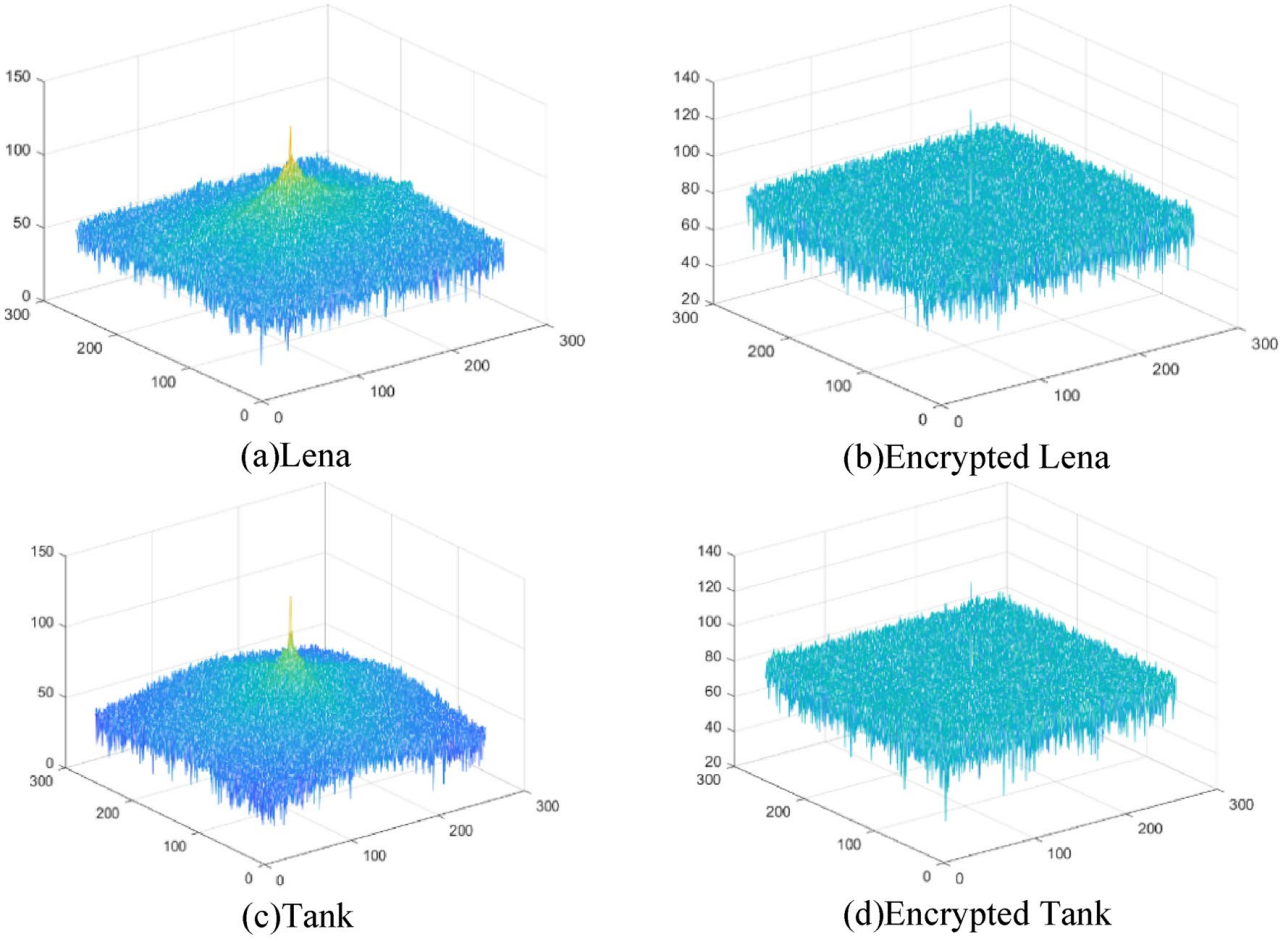
Spectra of the original and encrypted images.

The statistical properties of the original images and the corresponding ciphertext images are depicted in Fig. 13, showcasing their Fourier spectra. The amplitude of the spectrum is uniformly distributed following the encryption process. This observation suggests that information leakage is minimal, demonstrating that the proposed method is capable of withstanding spectrum attacks.

### The simulation of the proposed algorithm on IBM Q platform

This section demonstrates the implementation of the proposed algorithm on the IBM Q platform. IBM provides access to a range of real quantum devices and simulators. These devices are accessible and can be used through Qiskit, an open source quantum software development kit, and IBM Q Experience, and they provide a virtual interface for coding quantum computers. For more details on the IBM Q platform, please refer to the previous work^51^.

**Figure 14:**
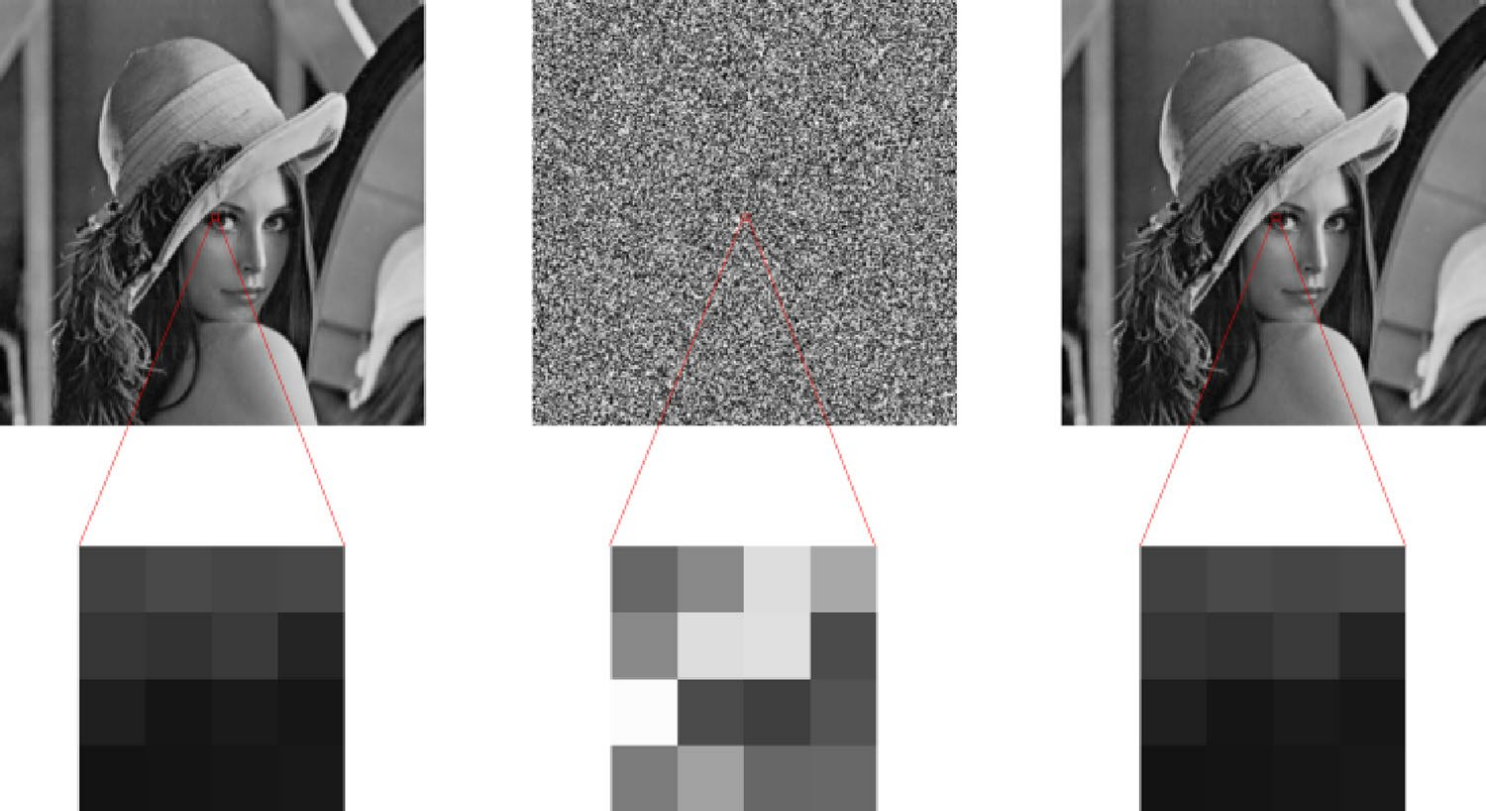
The image output by the quantum simulator.

In order to reduce the influence of randomness, the parameter is set to 8192. The image output by the quantum simulator is shown in the Fig. 14. The entire image encoded into qubits can be encoded as:

38 $$ \left| {Y_{7}  \ldots Y_{0} } \right\rangle  \otimes \left| {X_{7}  \ldots X_{0} } \right\rangle  \otimes \left| {C_{{YX}} } \right\rangle   $$

For the convenience of display, this article only gives the image example in the center of the image, the corresponding position of the binary code is

39 $$  \left| {100000Y_{1} Y_{0} } \right\rangle  \otimes \left| {100000X_{1} X_{0} } \right\rangle  \otimes \left| {C_{{YX}} } \right\rangle   $$

In order to make the measurement result more concise and intuitive, we only measure $\left| {Y_{1} Y_{0} } \right\rangle \left| {X_{1} X_{0} } \right\rangle \left| {C_{{YX}} } \right\rangle$ total 12 qubits under formula $\left| {Y_{7}  \ldots Y_{2} } \right\rangle  = \left| {100000} \right\rangle ,\left| {X_{7}  \ldots X_{2} } \right\rangle  = \left| {100000} \right\rangle$.

**Figure 15:**
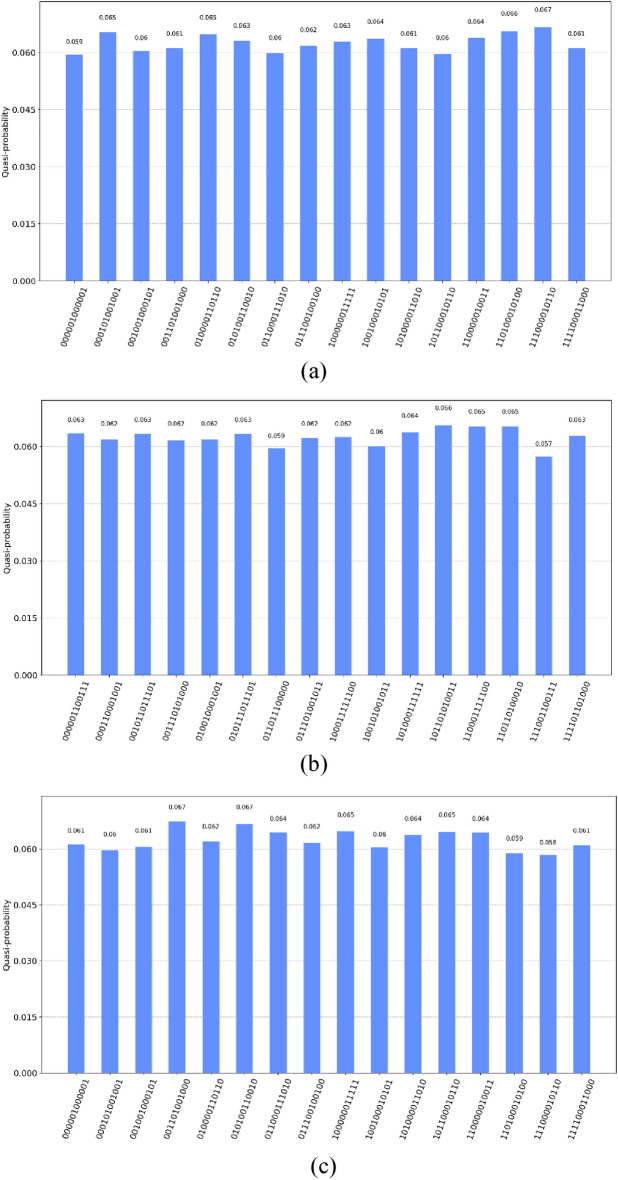
(**a**) 4 × 4 image probability histogram before encryption, (**b**) 4 × 4 image probability histogram after encryption, (**c**) 4 × 4 image probability histogram after decryption.

**Figure 16:**
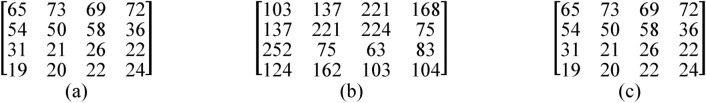
(**a**) The image matrix representation of the original image decoded, (**b**) The image matrix representation of the encrypted image decoded, and (**c**) The image matrix representation of the decrypted image decoded.

First, we encode the original image using quantum bits. Then, we measure the encoded initial image and obtain the measurement results are shown in the Fig. 15a. The corresponding image matrix obtained is shown in Fig. 16a. The measurement results are the same as the initial image, which proves the correctness of image coding in quantum image.

The quantum state result $\left| {Y_{1} Y_{0} } \right\rangle \left| {X_{1} X_{0} } \right\rangle \left| {C_{{YX}}^{\prime } } \right\rangle$ obtained after the encryption processing of the above algorithm is measured on the original image, and the measurement results are shown in the Fig. 15b. The corresponding classical matrix is shown in Fig. 16b.

The quantum state result $\left| {Y_{1} Y_{0} } \right\rangle \left| {X_{1} X_{0} } \right\rangle \left| {C_{{YX}}^{{\prime \prime }} } \right\rangle$ obtained after the decryption processing of the above algorithm is measured on the encrypted image, and the measurement results are shown in the Fig. 15c. The corresponding classical matrix is shown in Fig. 16c. The measurement results demonstrate that the obtained image matrix is identical to the original image matrix, providing evidence for the feasibility of quantum image coding.

## Conclusion

In this paper, we propose a novel image encryption algorithm utilizing Arnold transform and bit-plane chaotic mapping system. To enhance security, we adopt unequal Arnold transform parameters and the initial values of Lorenz chaotic map as keys. This not only simplifies key transmission but also provides an infinite key space to resist brute force attacks. Additionally, our proposed algorithm is capable of encrypting images of various sizes. We conducted extensive simulations and performance analyses to verify the effectiveness of our method. These analyses include histogram analysis, key space analysis, and robustness analysis. Results demonstrate that the algorithm is secure and reliable for image encryption purposes. Furthermore, by conducting simulation experiments using Qiskit, we successfully validate the correctness and feasibility of our quantum image encryption algorithm.

## Data Availability

The data used in this paper are available from the corresponding author upon request.
